# Calixarene-modified albumin for stoichiometric delivery of multiple drugs in combination-chemotherapy

**DOI:** 10.7150/thno.72559

**Published:** 2022-05-01

**Authors:** Ying Wang, Zhanzhan Zhang, Xinzhi Zhao, Lina Xu, Yadan Zheng, Hua-Bin Li, Dong-Sheng Guo, Linqi Shi, Yang Liu

**Affiliations:** 1 Key Laboratory of Functional Polymer Materials of Ministry of Education, College of Chemistry, Nankai University, Tianjin 300071, China.; 2 State Key Laboratory of Medicinal Chemical Biology, Nankai University, Tianjin 300071, China.; 3 State Key Laboratory of Elemento-Organic Chemistry, Nankai University, Tianjin 300071, China.; 4 College of Veterinary Medicine, Northeast Agricultural University, Harbin 150030, China.

**Keywords:** combination chemotherapy, co-delivery, hypoxia-responsive, host-guest interaction, stoichiometric

## Abstract

**Rationale:** In combination chemotherapy, the molar ratio of drugs is a critical parameter that determines the synergistic effects. However, most co-delivery vectors are incapable of maintaining the optimal molar ratio of drugs throughout the delivery process. Herein, a calixarene-modified albumin (CaMA), which can co-deliver multiple drugs with precise control of the drug ratio, is presented.

**Methods:** CaMA was prepared by chemically conjugating multiple sulfonate azocalix[4]arenes (SAC4A) onto the surface of bovine serum albumin (BSA). The precise drug loading and synchronous drug release were measured using fluorescence spectroscopy. Mouse tumor cell 4T1 and 4T1-bearing mice were used to evaluate the combined effects of mitomycin C (MMC) and doxorubicin (DOX) *in vitro* and *in vivo*.

**Results:** With multiple hypoxia-responsive calixarenes conjugated onto a single albumin molecule, CaMA achieved precise drug loading and synchronous release of multiple drugs into the tumor microenvironment. This unique drug loading and release mechanism ensures that CaMA maintains the drug ratio from the initial drug loading to the release site, providing a solid foundation for multi-drug combination therapy with the goal of achieving predictable therapeutic outcomes *in vivo*. The delivery of the model drug combination MMC and DOX at a prescreened ratio via CaMA achieved significantly enhanced tumor suppression and reduced systemic toxicity.

**Conclusions:** This stoichiometric delivery feature makes CaMA a powerful tool for the development of combination chemotherapy and personalized medications for cancer treatment.

## Introduction

Combination drug therapy is a common practice in cancer treatment to improve therapeutic outcomes by exploiting the different toxicities of multiple drugs 1-7. In this form of therapy, the molar ratio of the combined drugs is a critical parameter that determines the synergistic effects 8-12. However, the optimal molar ratio of drugs identified *in vitro* is difficult to maintain after delivery to the targets *in vivo* due to the differences in drug biodistribution and pharmacokinetics (PK) 13, which impairs the synergistic effects of the drug combination and hinders its clinical application. To date, several nano-drug delivery systems (nanoDDS), such as polymer micelles 14-16 and liposomes 17, have been investigated for use in the co-delivery of multiple drugs 18-25. Although these strategies ensure a consistent biodistribution and PK of the loaded drugs 8, 11, 26, 27, precise control of the drug ratio delivered to tumors remains challenging due to the lack of an accurate loading strategy for multiple drugs (physical absorption/embedment is still the major method for drug loading in most co-delivery nanoDDS), as well as the potential for leakage during transportation 23. Moreover, imprecise loading and drug leakage may lead to “batch-to-batch” variations in preparation and individual differences in drug delivery, resulting in an unpredictable therapeutic efficacy in cancer treatment 28, 29. To address this issue, covalent conjugation strategies (i.e., drug-to-carrier 13, 30 or drug-to-drug 11, 12, 31, 32) via stimuli-responsive bonds have been developed 24, 30, 33. However, the specific groups required for chemical conjugation are not always available for many chemotherapeutic drugs, and tedious synthesis and purification processes limited the fine-tuning of the drug ratio 34. Therefore, a co-delivery vector that allows for: i) precise loading of multiple drugs, ii) easy tuning of the molar ratio of loaded drugs, and iii) stoichiometric co-delivery of drug combinations to the tumor is in urgent demand for efficient combination chemotherapy.

In the past few decades, macrocyclic molecules (i.e., pillararenes 35-38, cucurbiturils 39, 40, calixarenes 41, 42 and cyclodextrins 43) have been extensively investigated in drugs delivery to enhance stability, improve solubility, and reduce the side effects of drug 44-47. These macrocyclic hosts complex with drug molecules through host-guest interactions with a characteristic binding affinity and defined stoichiometry (mostly 1:1) 48, 49. This unique drug loading mechanism allows for the prediction of the amount of drug loaded into the macrocyclic hosts according to their initial concentrations and characteristic binding affinities, providing a solid foundation for the precise control of drug loading 28, 41. However, the 1:1 stoichiometry between macrocyclic and drug molecules restricts the simultaneous loading and co-delivery of multi-drug 28. Therefore, for an efficient macrocyclic-based delivery system for combination chemotherapy, it is essential to develop innovative strategies that can (i) overcome the 1:1 stoichiometric limitation and achieve precise loading of multiple drugs, (ii) effectively target tumor tissues, and (iii) maintain the drug ratio after delivery for optimal synergistic effects of the drug combination.

**Scheme 1:**
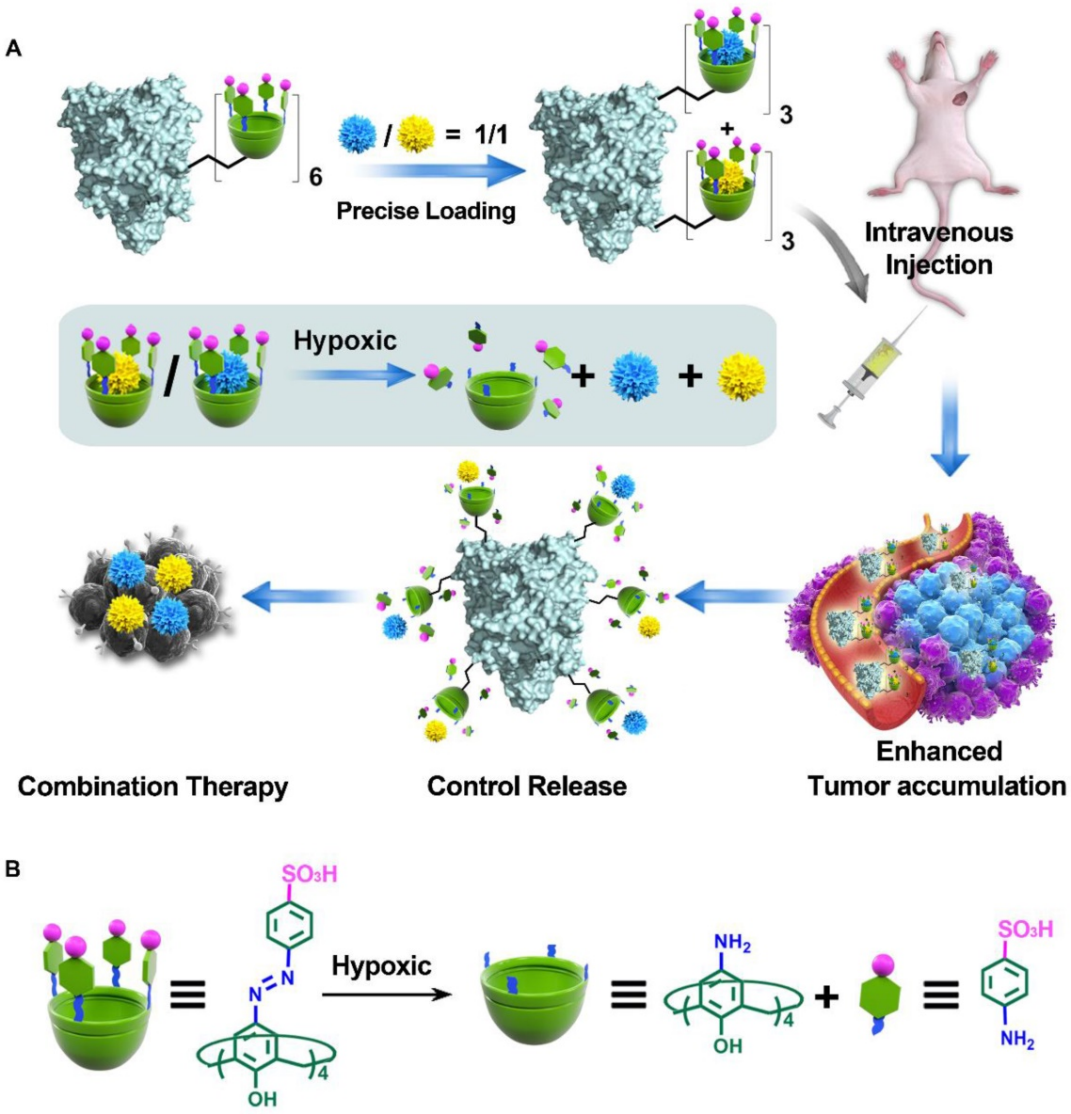
Illustration of CaMA for combination chemotherapy. **(A)** Illustration of CaMA for stoichiometric co-delivery of drug combinations. **(B)** Mechanism of SAC4A degradation under hypoxic condition.

Herein, we developed a calixarene-modified albumin (CaMA) as a stoichiometric co-delivery system for the simultaneous delivery of multiple drugs with a precise control of the drug ratio. As illustrated in **Scheme 1**, CaMA is prepared by chemically conjugating multiple sulfonate azocalix[4]arenes (SAC4A) onto the surface of bovine serum albumin (BSA). BSA is employed as the structural basis of CaMA due to its nanoscale particle size, stable physicochemical structure, high biocompatibility, and wide availability. SAC4A, which is a stimuli-responsive calixarene that degrades in the hypoxic tumor microenvironment (TME), serves as the functional unit of CaMA to load and control the release of drug molecules. With multiple SAC4As on the surface, CaMA can load multiple drug molecules within one nanostructure. More importantly, more than one type of drug molecule can be loaded into CaMA, and the drug ratio is able to precisely control by adjusting the initial drug concentrations calculated from their characteristic binding affinities. In blood circulation or normal tissue, the strong binding affinity between CaMA and the loaded drugs prevents the drugs from undesired leakage. Upon reaching the TME, the binding affinity decreases as SAC4A degrades, resulting in the rapid and simultaneous release of all drugs. All these characteristics of CaMA, including predictable drug loading ratio, minimal drug leakage during the transportation, enhanced tumor accumulation and simultaneous release of all drugs in response to TME, ensure the stoichiometric delivery of multiple drugs to *in vivo* target. In this study, doxorubicin (DOX) and mitomycin C (MMC), which are commonly used in clinical chemotherapy, were chosen as the model drug combination. The ratio of DOX to MMC to achieve optimal anti-cancer efficacy was first screened *in vitro*. By loading DOX and MMC in the pre-screened ratio, CaMA achieved stoichiometric delivery of the drug combination to the tumors, resulting in significantly enhanced tumor suppression and reduced toxicity compared with conventional cocktail therapy. This stoichiometric delivery ability allows CaMA to rapidly translate optimal drug combinations prescreened* in vitro* into *in vivo* therapeutic benefits, providing a powerful tool for the development of combination chemotherapies and personalized medications for cancer treatments.

## Methods

### Preparation of CaMA

For the synthesis of CaMA, SAC4A was first reacted with 1-bromo-2,3-epoxypropane to obtain SAC4A-epoxy. Briefly, 400 mg Na_2_CO_3_ (100 mg/mL) and 1-bromo-2,3-epoxypropane (1 g, 7.3 mmol) were added in sequence to a solution of 35 mg SAC4A (28 μmol) in DMF (4 mL) and stirred for 24 h at room temperature. After this reaction, the insoluble Na_2_CO_3_ was first removed by centrifugation and unconjugated 1-bromo-2,3-epoxypropane was then removed by precipitation in a large quantity of diethyl ether, following by collecting the precipitation and drying it in vacuo to obtain the SAC4A-epoxy. CaMA was then prepared by conjugating the SAC4A-epoxy onto BSA via a reaction between the amino groups of BSA and the epoxy groups of SAC4A-epoxy. Briefly, 20 mg BSA (0.3 μmol) and 3.75 mg SAC4A-epoxy (3 μmol) were added to 5 mL Na_2_CO_3_ buffer (100 mM) and stirred for 24 h at room temperature. The product was isolated by dialyzing against water (MWCO 10000), followed by ultrafiltration (MWCO 30000) and desalination with desalting columns (MWCO 7000). The successful preparation of CaMA was confirmed by MALDI-TOF.

### Drug release under cellular hypoxic condition

The hypoxia-triggered drug release behavior of CaMA was observed by CLSM. Briefly, 4T1 cells were first seeded overnight in confocal imaging chambers (1 × 10^5^ cells/well) before treating with CaMA-DOX (8/8 μM). After 6 h's incubation, the culture medium was replaced with fresh medium, and the cells were incubated for another 18 h under hypoxic or normoxic condition, respectively. The cells were then rinsed and stained with DAPI for CLSM image.

### Precise drugs loading capability of CaMA

To investigate the capability of CaMA in precisely loading of the drugs, CPT and DOX were employed as the model drugs. The concentration of CaMA (determined by SAC4A) was fixed at 180 μM, the total concentration of CPT and DOX was fixed at 150 μM, and the feeding concentrations of CPT and DOX were calculated according to the expected molar ratio and *K*a. Specifically, the concentrations of DOX and CPT were prepared according to the following ratio: 1 : 5 = 21.5 μM : 125 μM, 1 : 2 = 42.5 μM : 100 μM, 1 : 1 = 75 μM : 88.24 μM, 2 : 1 = 100 μM : 58.82 μM and 5 : 1 = 125 μM : 39.41 μM. All mixtures of DOX, CPT and CaMA were shaken for 30 min at room temperature, followed by removing unloaded drugs by ultrafiltration centrifugation (MWCO = 3 kDa). Unloaded DOX and CPT in the filtrate were measured with fluorescence spectrometer at *λ*_ex_ = 497 nm and *λ*_ex_ = 365 nm.

### Synchronous release of loading drugs

To investigate the synchronous release capabilities of CaMA, DOX and CPT were loaded into CaMA at molar ratios ranging from 0.2:1 to 2:1 to form CaMA-DC (0.2:1), CaMA-DC (0.5:1), CaMA-DC (1:1), CaMA-DC (2:1). Specifically, the concentration of CaMA was fixed at 3.2 μM and the total concentration of DOX and CPT was fixed at 3.0 μM, therefore, CaMA-DC (0.2:1) = 0.5 μM : 2.5 μM, CaMA-DC (0.5:1) = 1.0 μM : 2.0 μM, CaMA-DC (1:1) = 1.5 μM : 1.5 μM, CaMA-DC (2:1) = 2.0 μM : 1.0 μM. CaMA-DC at different ratios were then incubated with DT-diaphorase/NADPH and the release of DOX and CPT were continuously monitored during the incubation period.

### Combination index analysis of DOX and MMC

A cell viability-based assay was used to determine the combined index (CI) of DOX and MMC. Briefly, different concentrations of DOX and MMC (16, 8, 4, 2, 1, 0.5, 0.25, and 0.125 μM) and combinations of different ratios (4:1, 2:1, 1:1, 1:2, 1:4) were used to treat 4T1 cancer cells, and their cytotoxicity against 4T1 cells was assayed with CCK-8. Afterwards, the CI for DOX and MMC was conducted using CompuSyn software based on the Chou and Talalay method. CI values > 1, = 1, and < 1 indicate antagonistic, additive, and synergistic effects of the drug combination. The best-fit CI values at IC50 were used to show and compare the synergy of drug combination at different ratios.

### Anti-tumor efficacy of DOX and MMC

Female BALB/c mice were used to investigate the synergistic anti-tumor effects of MMC and DOX. First, 1×10^6^ 4T1 cancer cells were subcutaneously injected into the left mammary fat pad of mice to establish the tumor-bearing mice model. Then the mice were randomly divided into seven groups (*n* = 6) once the tumor volumes had reached approximately 100 mm^3^. 200 μL of PBS, CaMA (320 μM), DM (molar ratio 1:1, 150 μM/150 μM), CaMA-DOX (320 μM/300 μM,), CaMA-MMC (320 μM/300 μM), CaMA-DM-med (320 μM/60 μM/240 μM), and CaMA-DM-opt ((320 μM/150 μM/150 μM) were then intravenously injected into the mice every two days for a total of 3 doses. PBS-treated mice were used as negative control. The volume of tumors were consciously monitored for 25 days, and calculated on the basis of the formula: *V* = (*W*^2^ × *L*)/2, where W represents the shortest dimension and L represents the longest dimension.

## Results and Discussion

### Structure characterization of CaMA

**Figure 1:**
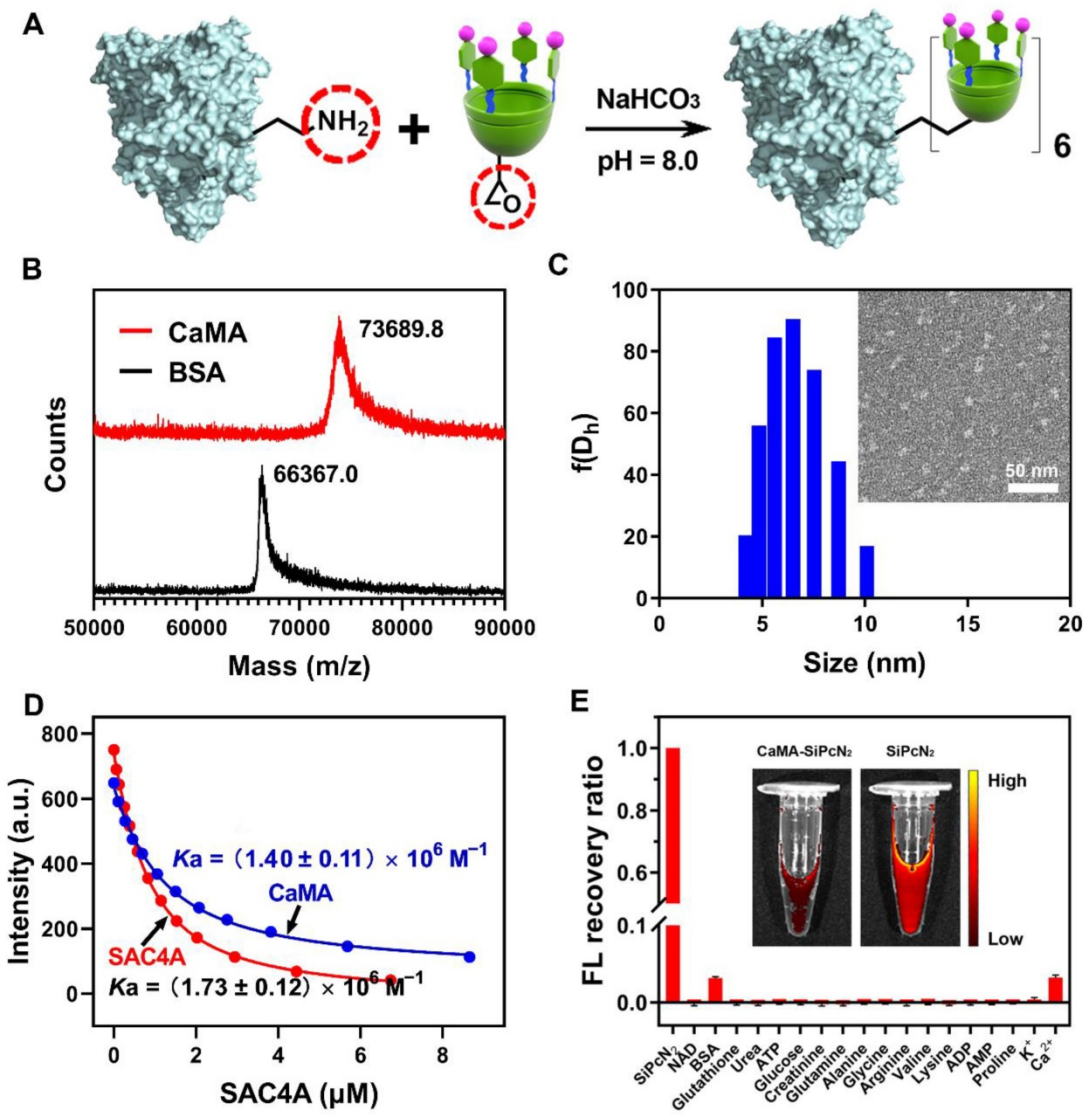
** Structural characterization of CaMA. (A)** The synthesis of CaMA. **(B)** MALDI-TOF spectrum of BSA and CaMA. **(C)** Size distribution and TEM image of CaMA. **(D)** Fluorescence titration curves of RhB with CaMA and SAC4A fitted according to the 1:1 binding stoichiometry. **(E)** Fluorescence (FL) recovery ratio of CaMA-SiPcN_2_ in the presence of biological species in blood, and the fluorescence image of CaMA-SiPcN_2_ and SiPcN_2_ in MS (inset).

To prepare CaMA, a hypoxia-responsive macrocyclic host, SAC4A was first synthesized as the functional unit to precisely load and responsively release of drugs 50. In the design of SAC4A, aminocalix[4]arene (NH_2_C4A) was used as macrocyclic scaffold. By modifying the upper rim of NH_2_C4A with 4-sulfobenzenediazonium chloride, SAC4A obtained a cavity to enhance the binding affinity to guests attributed to its deeper cavitand and the formation of salt bridges between the sulfonate and cationic groups of the guest molecules (drugs, probes, etc.). After obtaining SAC4A, CaMA was synthesized by conjugating SAC4A onto BSA. This was achieved by first reacting SAC4A with 1-bromo-2,3-epoxypropane to obtain SAC4A-epoxy, followed by conjugating SAC4A-epoxy onto BSA through a reaction between the amino groups of BSA and the epoxy groups of SAC4A-epoxy (**Figure 1A** and **Figure S1**). The successful conjugation of SAC4A onto BSA was confirmed by MALDI-TOF analysis, in which the characteristic peak of CaMA was detected with a molecular weight of 73689.8 Da (**Figure 1B**). The average conjugated SAC4A molecule per BSA was calculated to be 5.86 according to the MALDI-TOF spectrum. Zeta potential analysis further confirmed the conjugation of SAC4A onto BSA, with the decrease in zeta potential from -15.6 ± 0.21 mV of BSA to -21.2 ± 0.48 mV of CaMA. Dynamic light scattering (DLS) measurements and transmission electron microscopy (TEM) observations indicated that CaMA has a uniform nanomorphology with a diameter of around 6.5 ± 0.78 nm (**Figure 1C**).

It is necessary for the SAC4A of CaMA to maintain the drug loading capability after chemical conjugation onto BSA for an efficient combination drug therapy. Rhodamine B (RhB), DOX, CPT and MMC were employed as the reporter, and their binding affinity (*K*_a_) to the SAC4A of CaMA and free SAC4A were determined by fluorescence titration. As shown in **Table S1**, **Figure 1D** and **Figures S2-9**, slightly decreased binding affinities to RhB, DOX, CPT and MMC were observed from the SAC4A of CaMA compared to free form of SAC4A, suggesting the uncompromised drug loading capability of the SAC4A after conjugating to BSA and the potential drug loading capability of CaMA. To demonstrate the capability of CaMA as a co-delivery vector for combination drug therapy, common anticancer drugs, including camptothecin (CPT), DOX and MMC were employed and their binding affinities to CaMA were determined. As shown in the results from fluorescence titration (**Figures S4, S6 and S8**), CaMA exhibited strong binding affinities to CPT ((1.85 ± 0.35) × 10^5^ M^-1^), DOX ((9.98 ± 0.16) × 10^6^ M^-1^), and MMC ((8.47 ± 0.71) × 10^5^ M^-1^) with a 1:1 stoichiometry. This high binding affinity guaranteed the quantitative loading of these drugs. For example, when the concentration of CaMA was 300 μM, the drug loading efficacy of CaMA can be calculated as 98% for DOX, 94% for MMC and 87% for CPT (see Supporting Information for the detailed calculation). Considering that one CaMA possesses multiple SAC4As, the strong binding affinities of these drugs offer CaMA great potential for achieving the precise loading of drug combinations by control the initial drug concentrations calculated from their characteristic binding affinities.

As a non-covalent co-delivery vector, it is essential for CaMA to avoid undesired payload leakage during blood circulation. For the verification, the major species existing in blood were employed to challenging the integrity the CaMA. A fluorescent probe, silicon (IV) phthalocyanine (SiPcN_2_), was loaded into CaMA to form CaMA-SiPcN_2_ (**Figure S10**), then the major species in blood were utilized to challenge the stability of CaMA-SiPcN_2_. Almost no fluorescence recovery was observed from CaMA-SiPcN_2_ after incubation with these biological species (**Figure 1E**), indicating the robustness of CaMA-SiPcN_2_ under physiological conditions. Furthermore, CaMA-SiPcN_2_ also maintained high stability in mouse serum (MS), in which negligible fluorescence signals were observed from CaMA-SiPcN_2_ in the presence of MS (**Figure 1E, inset**). A similar result was also observed from a similar stability study with CaMA-DOX complex (**Figure S11**). The anti-interference ability should be attributed to the strong host-guest interaction between CaMA and the guest molecules. This important feature of CaMA effectively avoids undesired drug leakage during blood circulation, thereby endowing CaMA with great potential for the stoichiometric co-delivery of multiple drugs to tumor.

### Hypoxia-responsive degradation of CaMA

**Figure 2:**
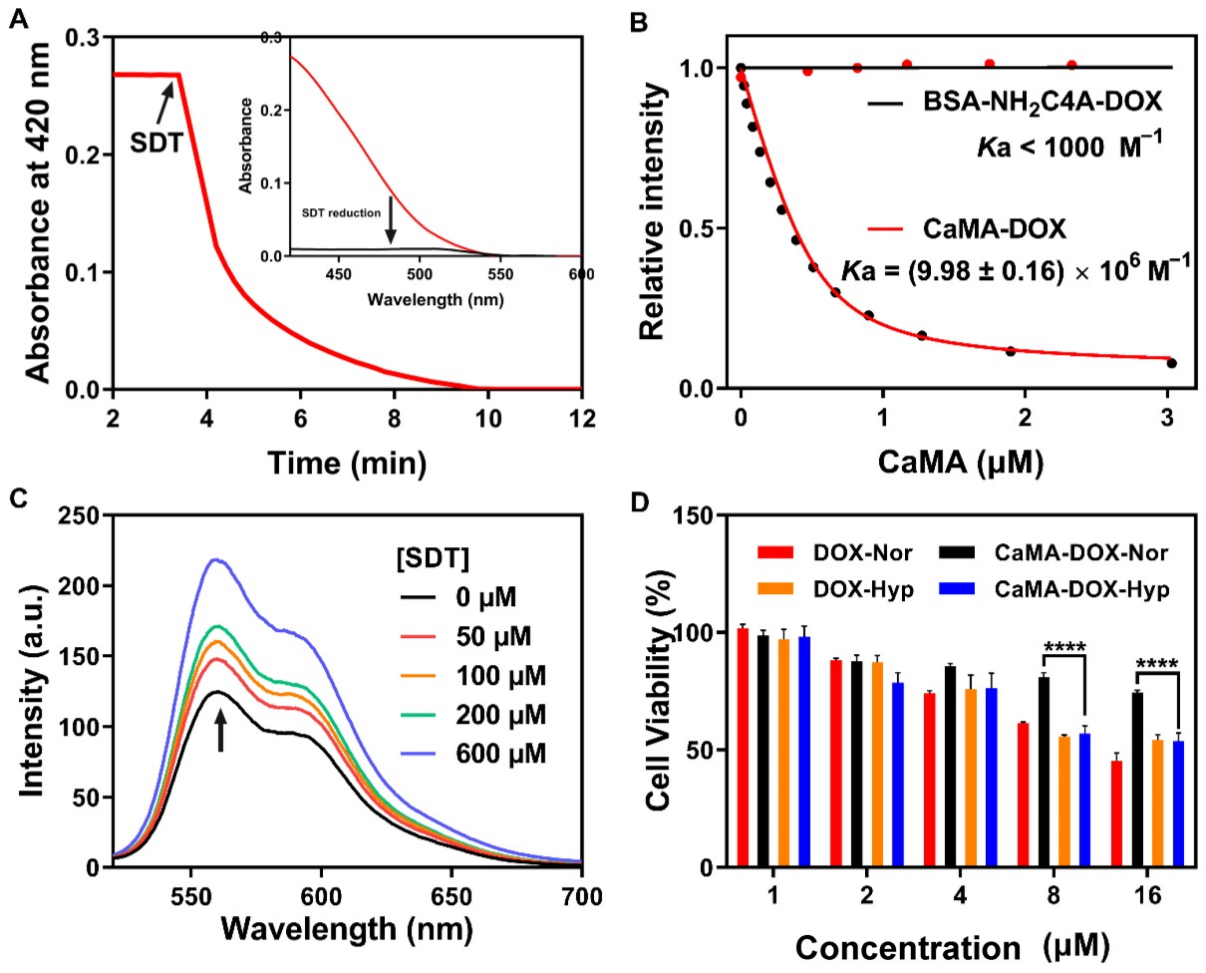
** Hypoxia-responsive degradation of CaMA. (A)** Absorbance changes for CaMA (5 µM) at 420 nm after adding SDT (2 µM), and the UV spectra of CaMA before and after SDT addition (inset). **(B)** Fluorescence titration curves of CaMA and BSA-NH_2_C4A with DOX, fitted according to the 1:1 binding stoichiometry. **(C)** Fluorescence spectra of CaMA-DOX after incubation with different concentrations of SDT (0-600 µM) in PBS for 30 min. λ_ex_ = 497 nm. **(D)** Cell viabilities of the DOX and CaMA-DOX treated cells under different conditions. 'Nor' stands for normoxic condition, 'Hyp' stands for hypoxic condition.

The ability to precisely confine drug release to only the tumor site is another requirement for CaMA in order to improve therapeutic outcomes with reduced side effects. To this end, SAC4A of CaMA was designed to be reduced to NH_2_C4A by the reductase present in the hypoxic TME, thereby significantly reducing its binding affinity to the loaded drugs and releasing them. To demonstrate the hypoxia-responsive capability of CaMA, sodium dithionite (SDT) was employed as a chemical mimic of reductase and incubated with CaMA. The absorbance was continuously monitored at 420 using a UV-vis spectrometer. The absorbance at 420 nm declined sharply and disappeared within 10 min after the addition of SDT (**Figure 2A**), indicating a rapid and complete reduction of the azo groups. Similar studies confirmed that CaMA could also be reduced by DT-diaphorase with NAD phosphate (NADPH) under hypoxic condition (**Figure S12**). Next, the binding affinities of CaMA to the drug molecules before and after reduction were studied using DOX as guest molecule. The binding affinity between DOX and reduced CaMA (BSA-NH_2_C4A) (*K*a < 1000 M^-1^) was four orders of magnitude lower than that of CaMA (*K*a = (9.98 ± 0.16) × 10^6^ M^-1^) (**Figures 2B**,** S4** and **S13**). This significantly reduced binding affinity indicates the potential of CaMA releasing loaded drugs after reduction. To demonstrate, CaMA-DOX was treated with different concentrations of SDT and the fluorescence intensity was recorded using a fluorescence spectrometer. The fluorescence intensity of DOX was gradually recovered as the concentration of SDT increased (**Figure 2C**), indicating the loaded drug was released from CaMA under hypoxic TME. The release behaviors of each drug also confirmed that CaMA was able to achieve effective drug release under hypoxic TME (**Figure S14**). Further studies by challenging CaMA-DOX with other factors commonly present in inflammatory tissues, including low pH (pH = 6.5), elevated levels of ATP (100 μM) and redox species (10 mM GSH, 100 μM H_2_O_2_), resulted in negligible fluorescence recovery (**Figure S15**), suggesting that the drug release of CaMA can only be triggered by the reductases. This unique controllable release mechanism of CaMA not only optimizes the bioavailability of the drugs, but also provides the necessary prerequisites for the stoichiometric co-delivery of multiple drugs to tumor.

Next, the capability for controlled drug release was further verified with cell-based studies. For this demonstration, CaMA-DOX was first incubated with mouse breast cancer cells (4T1) under normoxic and hypoxic conditions, and the release of DOX was observed with confocal laser scanning microscopy (CLSM). An obvious fluorescence signal of DOX (red) was observed from the CaMA-DOX treated cells under hypoxic condition rather than under normoxic condition (**Figure S16**), indicating the hypoxia-responsive release of DOX from CaMA-DOX. The cell viability assay further confirmed this result (**Figure 2D**), in which significantly lower cell viability was observed from the CaMA-DOX treated cells under hypoxic condition than those under normoxic condition. The significantly different cytotoxicity of CaMA-DOX under different conditions demonstrated the capability of CaMA in optimizing the anti-tumor effect and reducing the undesired toxicity of the loaded drugs, which is essential for CaMA to achieve optimal therapeutic outcomes against tumor without obvious side effects.

### Precise drug loading and synchronous drug release capability of CaMA

**Figure 3:**
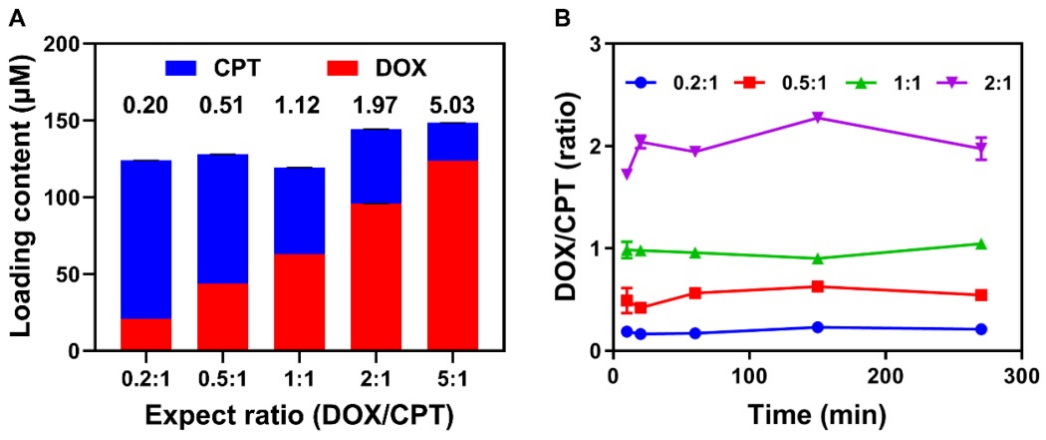
** Precise drug loading and synchronous drug release capability of CaMA. (A)** Loading contents and molar ratio of CPT and DOX in CaMA for different ratios. **(B)** The molar ratios of DOX to CPT released from CaMA-DC (DOX to CPT, 0.2:1, 0.5:1, 1:1 and 2:1) with the time of DT-diaphorase and NADPH added in PBS under hypoxic condition.

For efficient combination chemotherapy, the precise loading of drug combinations *in vitro* and the synchronous release of loaded drugs in tumor are critical for CaMA to ensure that the optimal drug ratio is maintained in the tumor. To investigate the precise drug-loading capability of CaMA, CPT and DOX were chosen as drug combinations. Five combinations of DOX and CPT with different expected ratios (0.2:1, 0.5:1, 1:1, 2:1 and 5:1) with a fixed total concentration of 150 μM were loaded into CaMA (denoted as CaMA-DC, details in the Supporting Information). The initial concentrations of DOX and CPT were calculated on the basis of their characteristic binding affinities. Drug loading was achieved by mixing CaMA, DOX, and CPT in pre-calculated concentrations, and the unloaded drugs were separated by filtration (MWCO = 3 kDa). To calculate the drug-loading content, the concentrations of the unloaded CPT and DOX were measured with fluorescence spectrometer. The molar ratio of DOX into CPT loaded in CaMA was almost the same as expected (0.2 vs 0.20, 0.5 vs 0.51, 1.0 vs 1.12, 2.0 vs 1.97, 5.0 vs 5.03) (**Figure 3A**). These results indicated the excellent capability of CaMA in precise loading of multiple drugs. More importantly, the molar ratio of the loaded drugs could be easily tuned by altering their feeding concentrations. Next, we studied the synchronous release of CaMA. CaMA-DCs at different ratios (DOX to CPT, 0.2:1, 0.5:1, 1:1 and 2:1) were incubated with DT-diaphorase/NADPH and monitored the fluorescence intensities of CPT and DOX continuously. The release of CPT and DOX increased gradually within the observed 240 min (**Figures S17-18**), indicating that DOX and CPT were effectively released from CaMA after reduction. Notably, the molar ratio of DOX to CPT released from CaMA-DC for each group maintained almost unchanged during the drug release and was consistent with the initial loading ratio (**Figure 3B**), suggesting the synchronous release of the multiple drugs and the ability to maintain the drug ratio after release.

### Tumor-targeting capability of CaMA

Subsequently, we studied the potential of CaMA for tumor-targeted delivery of drug combinations. In this study, SiPcN_2_ was employed as a fluorescence probe and loaded into CaMA to obtain CaMA-SiPcN_2_. Upon reaching the tumor tissues, SiPcN_2_ was released from CaMA-SiPcN_2_, leading to the recovery in fluorescence. For the demonstration, 4T1-bearing mice were administrated with CaMA-SiPcN_2_ and free SiPcN_2_ via tail-vein. At 48 and 72 h post-injection, tumors and major organs were collected for *ex vivo* observation. As shown in **Figures S19-20**, the tumors were clearly delineated by CaMA-SiPcN_2_ at 48 h post-injection, suggesting that SiPcN_2_ was successfully released from CaMA-SiPcN_2_. Moreover, the fluorescence intensity of the tumor from CaMA-SiPcN_2_ treated mice was significantly higher than free SiPcN_2_ treated, indicating the ability of CaMA to deliver the loaded drug to tumor tissues.

### Antitumor efficacy of CaMA-DM

**Figure 4:**
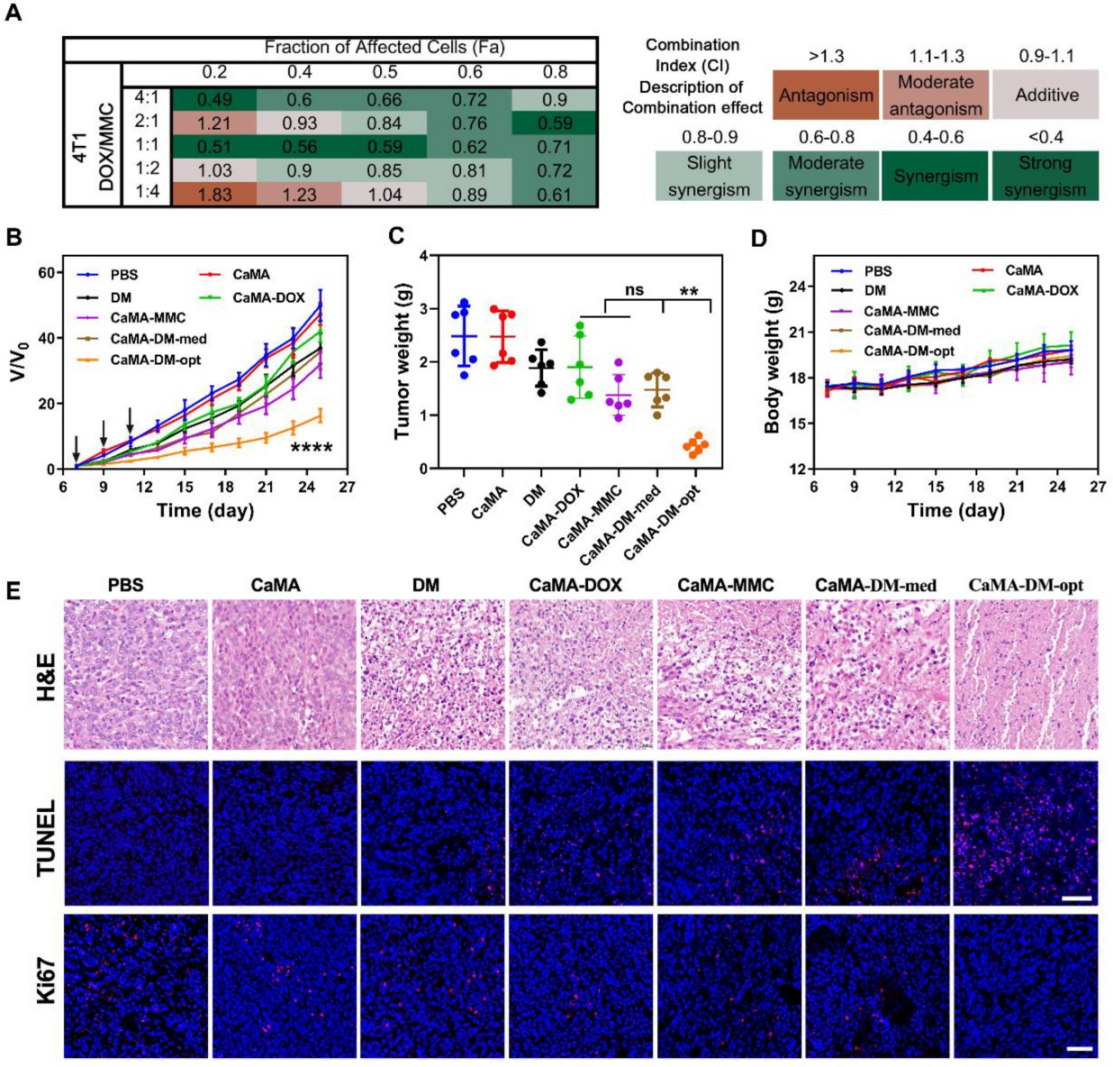
***In vivo***** antitumor efficacy of CaMA-DM. (A)** Combination index of DOX and MMC in 4T1 cells and corresponding color bar. **(B)** Growth curves of tumor from mice administrated with PBS, DM, CaMA, CaMA-DOX, CaMA-MMC, CaMA-DM-med and CaMA-DM-opt. **(C)** The tumor weights of mice from each group. **(D)** The changes in body weight of mice from each group. **(E)** H&E, TUNEL and Ki67 staining results of tumors from each group. Data are represented as mean ± s.e.m. (*n* = 6). The significant levels are ^**^P < 0.01, and ^****^P < 0.0001, analyzed by one-way ANOVA for (c) and two-way ANOVA for (b) with a Tukey's test.

Next, we evaluated the general applicability of CaMA as a co-delivery vector for effective combination chemotherapy. DOX and MMC were chosen as the drug combination owing to their non-overlapping cytotoxicity. Before applying to animals, the optimal ratio for the highest drug synergy of DOX and MMC on 4T1 cells was determined to be 1:1 with a cell-based assay on the basis of Chou-Talalay's method 51, 52 (**Figure 4A**). DOX and MMC at the optimal ratio (1:1) and the mediocre ratio (1:4) were then loaded into CaMA to form CaMA-DM-opt and CaMA-DM-med, followed by intravenously injecting into 4T1-bearing mice. PBS, CaMA (no drug), the mixture of DOX and MMC at ratio of 1:1 (DM), CaMA-DOX and CaMA-MMC were employed as the control and comparative groups for further investigation. The frequency of administration was once every two days for three doses (days 7, 9, and 11). The body weight and tumor volume were continuously monitored for 25 days. CaMA-DM-opt-treated mice exhibited the most effective tumor suppression than all other treatments (**Figure 4B-C**). In contrast, no significant difference in tumor suppression was observed between the groups treated with CaMA-DM-med, CaMA-DOX, and CaMA-MMC. These results are in keeping with the results from *in vitro* experiments, suggesting that CaMA can effectively maintain the molar ratio of drug combination throughout the delivery process. Additionally, the mice body weights in each group remained essentially unchanged within observed 25 days (**Figure 4D**). Further analysis of histopathological morphology of major organs (**Figure S21**) indicated that no obvious abnormalities or damages had been caused by CaMA-DM treatment. And a safety assessment by blood routine (**Figure S22**) indicated that no obvious inflammation was caused by CaMA. For the further investigation of the antitumor effects of CaMA-DM, the tumors were collected and sliced for hematoxylin-eosin (H&E), *in situ* terminal deoxynucleotidyl transferase dUTP nick end labeling (TUNEL), and Ki67 staining. As shown in the H&E image (**Figure 4E**), the tumor sections from the mice treated with CaMA-DM-opt exhibited classical apoptotic features, while negligible apoptosis signals were observed in other groups. Further staining with TUNEL and Ki67 also confirmed the same result, i.e., obvious apoptosis and significantly inhibited tumor proliferation were observed in tumor sections from the mice treated with CaMA-DM-opt. Considering that tumor suppression is closely related to the molar ratio of drug combinations, CaMA achieved to effectively translate the optimal molar ratio of the drug combination screened* in vitro* into *in vivo* tumor suppressions, providing a powerful tool for the development of safe and efficient combination chemotherapy and personalized medicine.

## Conclusions

In conclusion, we developed CaMA that achieves precise loading and stoichiometric delivery of multiple drugs for efficient combination chemotherapy. With multiple SAC4As on the surface, CaMA can load multiple drug molecules into one nanostructure through host-guest interactions. More importantly, the drug ratio is able to precisely control by adjusting the initial drug concentrations calculated from their characteristic binding affinities. Upon reaching the tumor, SAC4A of CaMA was degraded, allowing for the simultaneous release of all drugs. By employing DOX and MMC as a combination example, delivery of this drug combination at the pre-screened and optimal ratio via CaMA significantly enhanced tumor suppression with reduced toxicity compared with a traditional cocktail therapy. This stoichiometric delivery ability allows CaMA to rapidly translate optimal drug combinations pre-screened *in vitro* into *in vivo* tumor suppression, providing a powerful tool for the development of combination chemotherapies and personalized medications for cancer treatments.

## Supplementary Material

Supplementary materials and methods, figures and table.

## Abbreviations

CaMA: calixarene-modified albumin
MMC: mitomycin C
DOX: doxorubicin
PK: pharmacokinetics
nanoDDS: nano-drug delivery systems
BSA: bovine serum albumin
TME: tumor microenvironment
SAC4A: sulfonate azocalix[4]arene
NH_2_C4A: aminocalix[4]arene
DLS: dynamic light scattering
TEM: transmission electron microscopy
RhB: rhodamine B
CPT: camptothecin
MS: mouse serum
SiPcN_2_: silicon (IV) phthalocyanine
SDT: sodium dithionite
CLSM: confocal laser scanning microscope
H&E: hematoxylin-eosin
TUNEL: *in situ* terminal deoxynucleotidyl transferase dUTP nick end labeling
