# Supplementary material for: Calixarene-modified albumin for stoichiometric delivery of multiple drugs in combination-chemotherapy
Source: Theranostics. 2022 May 1;12(8):3747–57. doi: 10.7150/thno.72559 (PMC9131271; doi:10.7150/thno.72559)
Supplement: Supplementary file 1 — Supplementary materials and methods, figures and table. [file thnov12p3747s1.pdf]

Supporting Information

Calixarene-modified albumin for stoichiometric delivery of multiple drugs in combination chemotherapy

*Ying Wang[1,2#], Zhanzhan Zhang[1,2#], Xinzhi Zhao[1,2], Lina Xu[1,2], Yadan Zheng[1,2,4], Hua-Bin Li[1,3], Dong-Sheng Guo[1,3], Linqi Shi[1,2], and Yang Liu[1,2]**

[1] Key Laboratory of Functional Polymer Materials of Ministry of Education, College of Chemistry, Nankai University, Tianjin 300071, China.

[2] State Key Laboratory of Medicinal Chemical Biology, Nankai University, Tianjin 300071, China.

[3] State Key Laboratory of Elemento-Organic Chemistry, Nankai University, Tianjin 300071, China.

[4] College of Veterinary Medicine, Northeast Agricultural University, Harbin 150030, China.

* Correspondence and request for materials should be addressed to Yang Liu (yliu@nankai.edu.cn).

[#] These authors contributed equally to this work

Table of contents

1 Materials and methods 3

1.1 Materials 3

1.2 Instruments 3

1.3 Cell culture 4

1.4 Binding affinities of CaMA and SAC4A with guests 4

1.5 Data analysis of fluorescence titrations 4

1.6 The calculation of drug loading efficiency 5

1.7 Fluorescence responses of CaMA-SiPcN_2 and CaMA-DOX 6

1.8 *In vitro* cytotoxicity assays of CaMA-DOX and DOX 7

1.9 *In vivo* fluorescence imaging 7

1.10 The calculation of drug feeding ratio 8

1.11 Statistical Analysis 9

2 Supporting results and experimental data 10

Reference 21

1 Materials and methods

1.1 Materials

1-bromo-2,3-epoxypropane and glutathione were purchased from TCI. DT-diaphorase and rhodamine (RhB) were purchased from Sigma-Aldrich. Adenosine triphosphate (ATP) and sodium dithionite (SDT) were purchased from Across. Bovine serum albumin (BSA), alanine, glycine, arginine, valine, lysine and camptothecin (CPT) were purchased from Heowns. Adenosine diphosphate (ADP), nicotinamide adenine dinucleotide (NAD), adenosine monophosphate (AMP) and creatinine were purchased from Bidepharm. Doxorubicin (DOX) and mitomycin C (MMC) were purchased from Aladdin. Nicotinamide adenine dinucleotide phosphate (NADPH) was purchased from Solarbio. Silicon(IV) phthalocyanine chloride ($SiPcN_2$) was obtained from Frontier Scientific.

1.2 Instruments

Mass spectra were recorded on an AutoflexIII LRF200-CID (MALDI-TOF). Fluorescence spectra were recorded on a Hitachi F4600. UV-Visible spectra were acquired with a NanoDrop Onec (Thermo Scientific, USA). Dynamic light scattering (DLS) and zeta potential studies were performed on a Brookheaven ZETAPALS/BI-200SM (Brookheaven Instrument, USA). Transmission Electron Microscopy (TEM) measurements were performed on a Talos F200C electron microscope at an acceleration voltage of 200 kV. Cell viability was measured on a Tecan Spark plate reader. Flow cytometry analysis was performed on a BD LSR Fortessa flow cytometry. Confocal laser scanning microscope (CLSM) images were captured on TCS SP8 (Leica, Germany). Living imaging was performed with an IVIS Lumina imaging system (Caliper Life Sciences, USA).

1.3 Cell culture

The mouse breast cancer cells 4T1 was obtained from the American Type Culture Collection. 4T1 cells were cultured in Dulbecco's modified Eagle's medium with 10 % fetal bovine serum (FBS, v/v), 100 U/mL penicillin and 100 mg/mL streptomycin in an incubator (Thermo Scientific). For the normoxic condition, the 4T1 cells were incubated at 37 ˚C under an atmosphere of 5 % CO_2 and 90 % relative humidity. For the hypoxic condition, the 4T1 cells were incubated at 37 ˚C under an atmosphere of 94 % N_2, 5 % CO_2, 1 % O_2 and 90 % relative humidity.

1.4 Binding affinities of CaMA and SAC4A with guests

The direct fluorescence titration was used to determine their binding constants according to previously reported methods[1]. Briefly, the fluorescence intensity of dye solutions (0.5 μM) was firstly measured. After sequentially adding the host solutions, the changes of fluorescence intensity and the corresponding hosts concentrations were used to determine the association constant (K_a) of the host and guest. The data were fitted according to the 1:1 binding stoichiometry.

1.5 Data analysis of fluorescence titrations

The fitting of data from direct host-guest titrations was performed in a nonlinear manner[2], and the fitting modules were downloaded from the website of Prof. Nau's group (http://www.jacobs-university.de/ses/wnau) under the column of "Fitting Functions". It was introduced in detail as following:

For analyzing the host-guest direct titrations (equation **1**), we considered that a guest (G) formed a 1 : 1 host–guest complex with a host (H) at an association constant (K_a), which satisfied the

respective law of mass action relating to the equilibrium concentrations of free host, [H], free guest, [G], and host–guest complex [HG]. Also, the relationship between the total concentrations of host, $[H]_0$, and guest, $[G]_0$, and their equilibrium concentrations were introduced by the law of mass conservation (equation **2**). Here, $[G]_0$ was the initial concentration of guest as a known experimental parameter, which was kept constant in the titration process. Furthermore, equation **1** and **2-1** were employed to deduce equation **3**.

When the fluorescence titrations were performed, the intensity of fluorescence (F) corresponded to the combined intensity of the guest and the host–guest complex, which were described by their molar fractions (equation **4**). Both F_{HG} and F_G were the known experimental parameters, in which F_{HG} was the fluorescence intensity when all guests were complexed and F_G when they were not complexed. The equation **5** deduced by equation **2-2**, **3** and **4**, explained the relationship between K_a and variables ($[H]_0$) in fluorescence titrations. In the light of equation **5**, K_a was obtained by fitting the data of fluorescence intensity and total host concentration.

1.6 The calculation of drug loading efficiency

The drug loading efficiency was calculated according to the calculated tool of Nau' group (http://wernernau.user.jacobs-university.de/?page_id=243). In the calculation, the concentrations of both host and guests were 300 μM.

$$[\mathrm{H}]+[\mathrm{G}] \xrightleftharpoons{K_a} [\mathrm{HG}]$$

$$K_a = \frac{[\mathrm{HG}]}{[\mathrm{H}][\mathrm{G}]} \tag{1}$$

$$[\mathrm{G}] = [\mathrm{G}]_0 - [\mathrm{HG}] \tag{2-1}$$

$$[\mathrm{H}] = [\mathrm{H}]_0 - [\mathrm{HG}] \tag{2-2}$$

$$[\mathrm{HG}] = \frac{K_a[\mathrm{H}][\mathrm{G}]_0}{1+K_a[\mathrm{H}]} \tag{3}$$

$$F = \frac{[\mathrm{HG}]}{[\mathrm{G}]_0} F_{HG} + \frac{[\mathrm{G}]}{[\mathrm{G}]_0} F_G \tag{4}$$

$$F = F_{HG} + (F_G - F_{HG}) \frac{([\mathrm{G}]_0 - [\mathrm{H}]_0 - 1/K_a) - \sqrt{([\mathrm{G}]_0 - [\mathrm{H}]_0 - 1/K_a)^2 + 4[\mathrm{H}]_0[\mathrm{G}]_0}}{2[\mathrm{G}]_0} \tag{5}$$

1.7 Fluorescence responses of CaMA-SiPcN$_2$ and CaMA-DOX

Various biological species in blood were added separately to CaMA-SiPcN$_2$ (3/3 μM) and CaMA-DOX (5/5 μM) in PBS (10 mM, pH = 7.4) at 25 ˚C and stirred for 30 min to monitor the fluorescence intensity of SiPcN$_2$ and DOX. The fluorescence of SiPcN$_2$ or DOX alone was used as control. The biological species and their concertation used in these experiments were: nicotinamide adenine dinucleotide (NAD) 24 µM, bovine serum albumin (BSA) 10 µg/mL, glutathione 8.0 µM, urea 4.0 mM, adenosine triphosphate (ATP) 0.4 μM, glucose 5.0 mM, creatinine 80 μM, glutamine 0.5 mM, alanine 0.4 mM, glycine 0.3 mM, arginine 0.14 mM, valine 0.2 mM, lysine 0.2 mM, adenosine diphosphate (ADP) 0.1 µM, adenosine monophosphate (AMP) 10 nM, proline 0.2 mM, K^+ 4.5 mM and Ca^{2+} 2.5 mM. The concentrations of all these components refer to their concentrations in human blood.

1.8 *In vitro* cytotoxicity assays of CaMA-DOX and DOX

The anti-proliferation ability of CaMA-DOX and DOX was measured by CCK-8 assays. 4T1 cells were seeded into 96-well plates at a density of 1×10^4 cells/well and grown to 70-80 % confluence. The cells were then treated with CaMA-DOX and DOX of different concentrations (ranging from 1, 2, 4, 8, 16 μM). After incubating for 6 h under normoxic condition, the culture medium was replaced with fresh medium. The cells were then incubated for further 18 h under normoxic or hypoxic condition. PBS was used as the negative control. CCK-8 was mixed with culture medium at a volume ratio of 1:9 (freshly prepared) to afford CCK-8 working solution. After the incubation, the cells were rinsed twice with PBS, followed by the addition of 100 μL CCK-8 working solution and another 1.5 h's incubation. Quantification of the cell viability was achieved by measuring the absorbance with Tecan Spark plate reader (λ_{ex} = 450 nm).

1.9 *In vivo* fluorescence imaging

For the animal and the tumor model, female BALB/c mice at 6-8 weeks were purchased from Vital River Laboratory Animal Technology (Beijing, China). All animal studies were performed in accordance with the Regulations for the Administration of Affairs Concerning Experimental Animals (Tianjin, revised in June 2004) and adhered to the Guiding Principles in the Care and Use of Animals of the American Physiological Society, and were approved by the Animal Ethics Committee of Nankai University (Tianjin, China). All protocols within the study involving animals were approved by the Institutional Animal Care and Use Committee, Nankai University.

To establish the 4T1 tumor-bearing mouse model, 1×10^6 4T1 cancer cells were injected subcutaneously into the left mammary fat pad of BALB/c mice. The mice with tumor volumes

around 400 mm^3 were randomized into two groups and intravenously injected with 200 μL of $SiPcN_2$ and CaMA-$SiPcN_2$. Then the mice were sacrificed at 48 h and 72 h post-injection, and the tumor as well as major organs (heart, liver, spleen, lung, kidney) were collected for *ex vivo* imaging. Fluorescent images were taken by IVIS Lumina imaging system (Caliper Life Sciences, USA).

1.10 The calculation of drug feeding ratio

To calculate the feeding ratios according of drugs according to excepted ratios, we considered that the two drug guests G_1 and G_2 to form 1 : 1 host–guest complexes with the host (H) respectively with the association constant K_{a1} and K_{a2}, which satisfied the respective law of mass action relating to the equilibrium concentrations of free host, [H], free guests, $[G_1]$ and $[G_2]$, and host–guest complexes $[HG_1]$ and $[HG_2]$. Also, the relationship between the total concentrations of host, $[H]_0$, and guests, $[G_1]_0$, $[G_2]_0$, and their equilibrium concentrations were introduced by the law of mass conservation (equation **7**). Here, given $[H]_0 > [HG_1] + [HG_2]$, we assumed $[HG_1]$ and $[HG_2]$ were the excepted loading concentrations of guests and the concentrations of [H] was acquired as $[H] = [H]_0 - [HG_1] - [HG_2]$. The feeding ratios were acquired when the $[H]_0$, $[HG_1]$, $[HG_2]$, K_{a1}, K_{a2} and the excepted loading ratio (n) were determined.

$$[\mathrm{H}]+[\mathrm{G}_1] \underset{}{\overset{K_{a1}}{\rightleftharpoons}} [\mathrm{HG}_1] \tag{6-1}$$

$$[\mathrm{H}]+[\mathrm{G}_2] \underset{}{\overset{K_{a2}}{\rightleftharpoons}} [\mathrm{HG}_2] \tag{6-2}$$

$$K_{a1} = \frac{[\mathrm{HG}_1]}{[\mathrm{H}][\mathrm{G}_1]} \tag{6-3}$$

$$K_{a2} = \frac{[\mathrm{HG}_2]}{[\mathrm{H}][\mathrm{G}_2]} \tag{6-4}$$

$$[\mathrm{HG}_1]+[\mathrm{HG}_2] = [\mathrm{H}]_0 - [\mathrm{H}] \tag{7-1}$$

$$[\mathrm{HG}_1] = [\mathrm{G}_1]_0 - [\mathrm{G}_1] \tag{7-2}$$

$$[\mathrm{HG}_2] = [\mathrm{G}_2]_0 - [\mathrm{G}_2] \tag{7-3}$$

$$[\mathrm{HG}_1] = \frac{K_{a1}[\mathrm{H}][\mathrm{G}_1]_0}{1+K_{a1}[\mathrm{H}]} \tag{8-1}$$

$$[\mathrm{HG}_2] = \frac{K_{a2}[\mathrm{H}][\mathrm{G}_2]_0}{1+K_{a2}[\mathrm{H}]} \tag{8-2}$$

$$n = \frac{[\mathrm{HG}_1]}{[\mathrm{HG}_2]} \tag{9}$$

$$\frac{[\mathrm{G}_1]_0}{[\mathrm{G}_2]_0} = n\frac{K_{a2}(1+K_{a1}[\mathrm{H}])}{K_{a1}(1+K_{a2}[\mathrm{H}])} \tag{10}$$

1.11 Statistical Analysis

Data represent mean ± standard error of the mean (s.e.m.) from at least three independent experiments ($n \geq 3$) and the significance levels are *P<0.05, **P<0.01, ***P<0.001 and ****P<0.0001, analyzed by one-way or two-way ANOVA (when more than two groups were compared) with a Dunnett test. $P < 0.05$ or less was considered significant.

2 Supporting results and experimental data

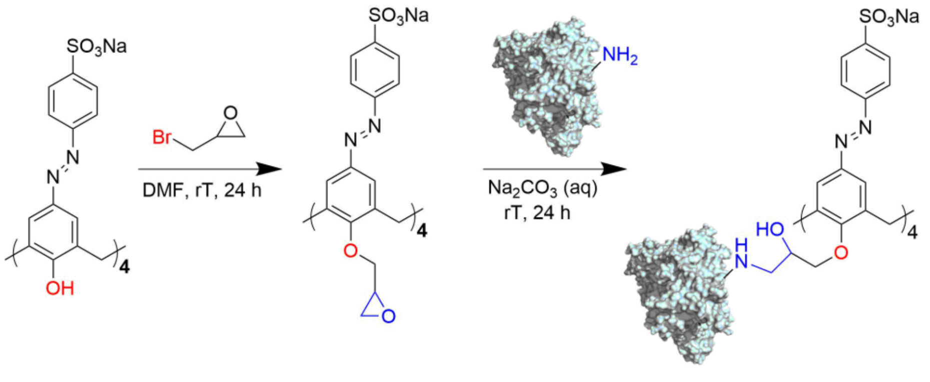

Figure S1. Synthesis routes of CaMA.

Table S1. The binding affinities of SAC4A and CaMA to drugs

	CaMA	SAC4A
RhB	(1.40 ± 0.11) × 10^6 M^{-1}	(1.73 ± 0.12) × 10^6 M^{-1}
DOX	(9.98 ± 0.16) × 10^6 M^{-1}	(1.18 ± 0.41) × 10^7 M^{-1}
CPT	(1.85 ± 0.35) × 10^5 M^{-1}	(3.79 ± 0.33) × 10^5 M^{-1}
MMC	(8.47 ± 0.71) × 10^5 M^{-1}	(8.71 ± 0.42) × 10^5 M^{-1}

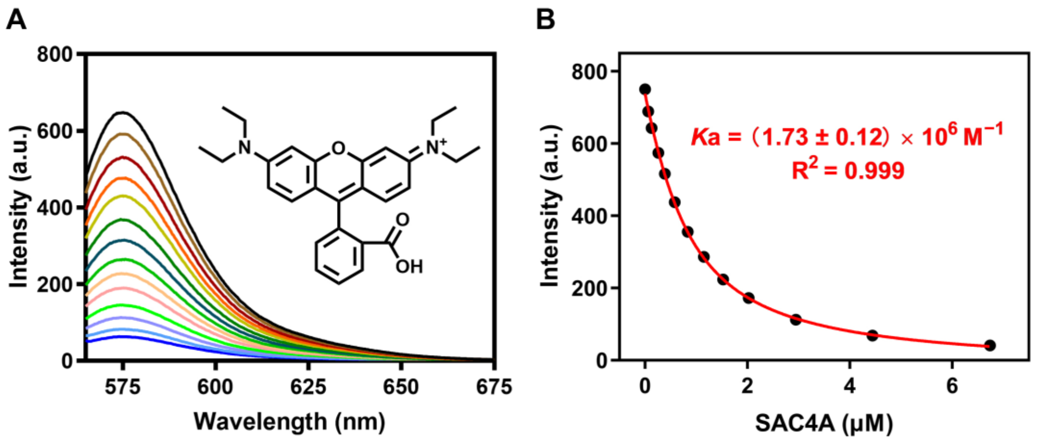

Figure S2. (A) Direct fluorescence titration of RhB (0.5 μM) with SAC4A (up to 6.74 μM) in PBS buffer at 25 ˚C, λ_{ex}= 554 nm; (B) The associated titration curve at λ_{em} = 575 nm was fitted according to a 1:1 binding stoichiometry.

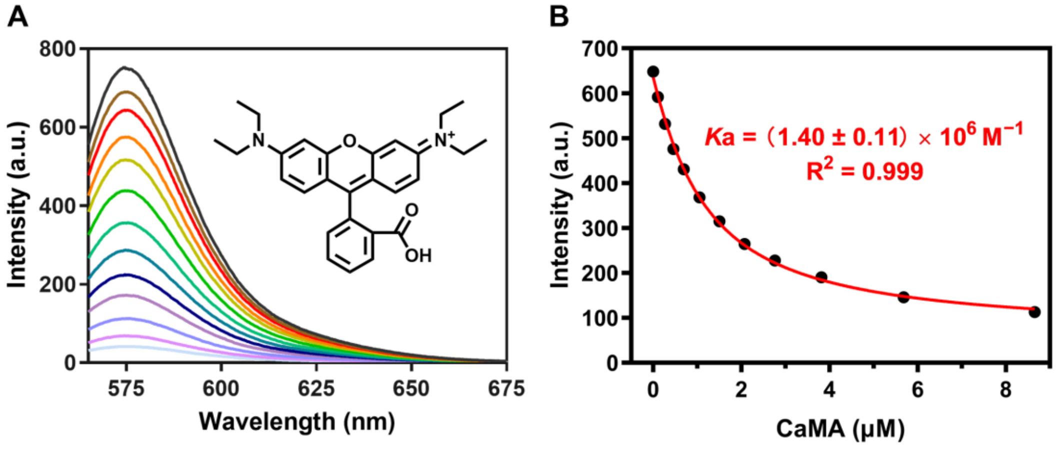

Figure S3. (A) Direct fluorescence titration of RhB (0.5 μM) with CaMA (up to 24.3 μM) in PBS buffer at 25 ˚C, λ_{ex} = 554 nm; (B) The associated titration curve at λ_{em} = 575 nm was fitted according to a 1:1 binding stoichiometry.

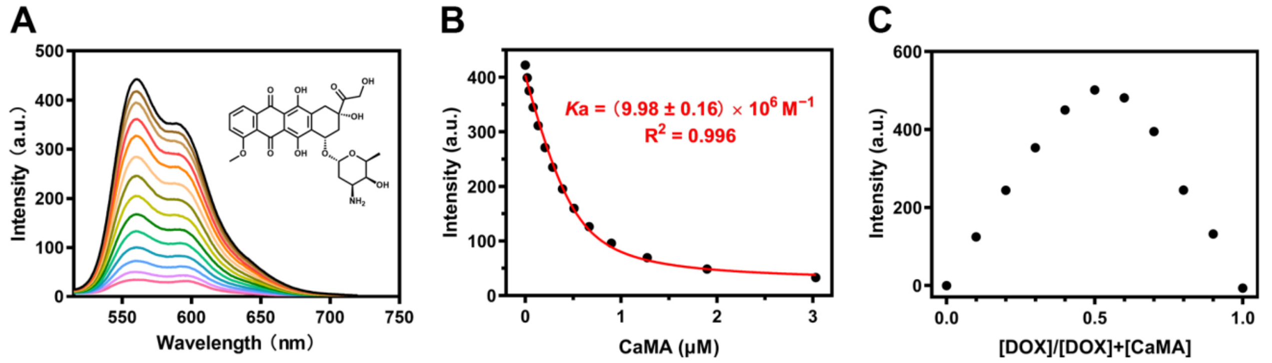

Figure S4. (A) Direct fluorescence titration of DOX (0.5 μM) with CaMA (up to 3.03 μM) in PBS buffer at 25 ˚C, λ_{ex}= 497 nm; (B) The associated titration curve at λ_{em} = 555 nm was fitted according to a 1:1 binding stoichiometry. (C) Job's plot for solutions of DOX and CaMA, [DOX] + [CaMA] = 2.0 μM.

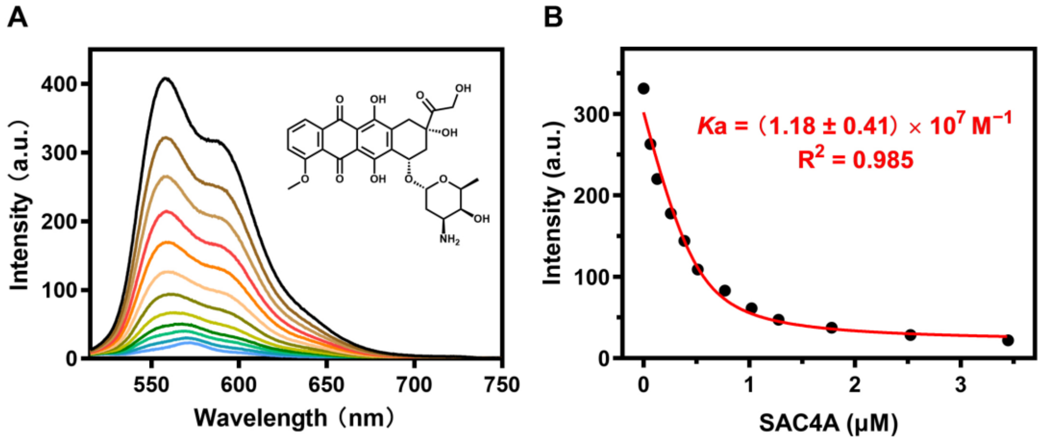

Figure S5. (A) Direct fluorescence titration of DOX (0.5 μM) with SAC4A (up to 3.45 μM) in PBS buffer at 25 ˚C, λ_{ex}= 497 nm; (B) The associated titration curve at λ_{em} = 555 nm was fitted according to a 1:1 binding stoichiometry.

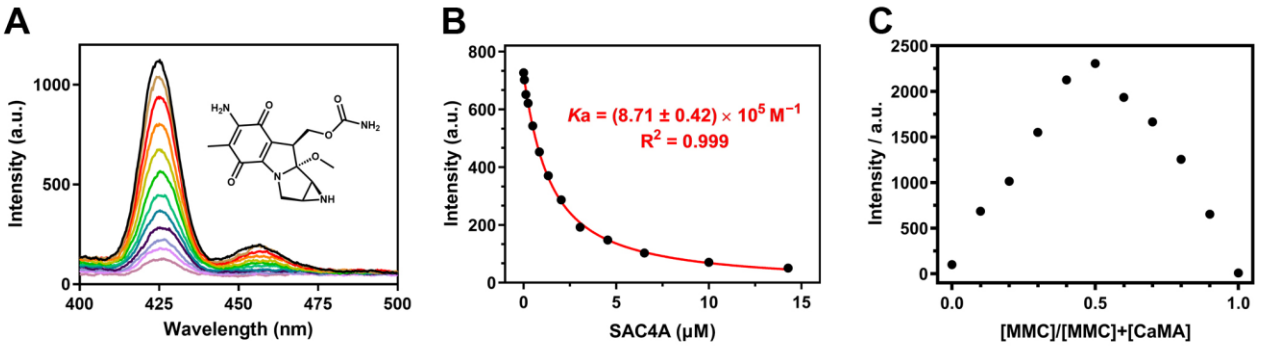

Figure S6. (A) Direct fluorescence titration of MMC (0.5 μM) with CaMA (up to 9.06 μM) in PBS buffer at 25 ˚C, λ_{ex}= 210 nm; (B) The associated titration curve at λ_{em} = 423 nm was fitted according to a 1:1 binding stoichiometry. (C) Job's plot for solutions of MMC and CaMA, [MMC] + [CaMA] = 2.0 μM.

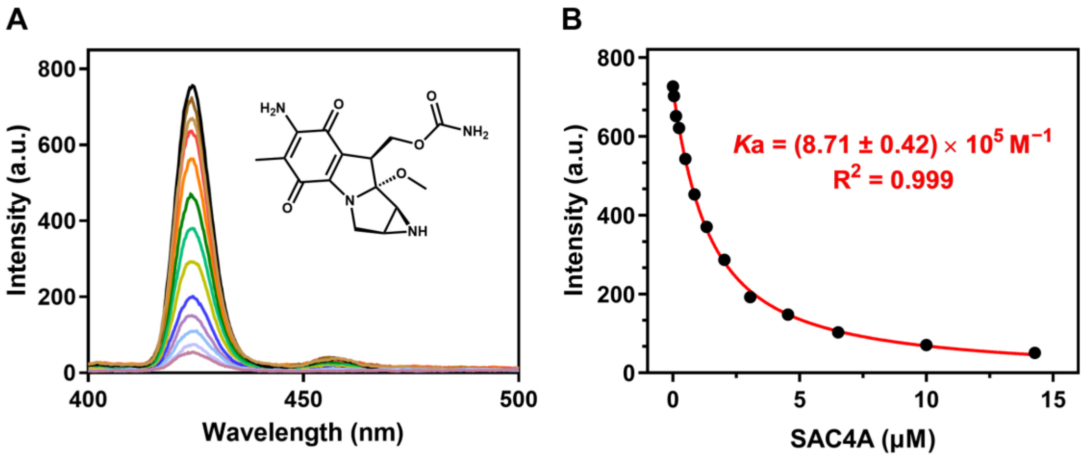

Figure S7. (A) Direct fluorescence titration of MMC (0.5 μM) with SAC4A (up to 14.29 μM) in PBS buffer at 25 ˚C, λ_{ex}= 210 nm; (B) The associated titration curve at λ_{em} = 423 nm was fitted according to a 1:1 binding stoichiometry.

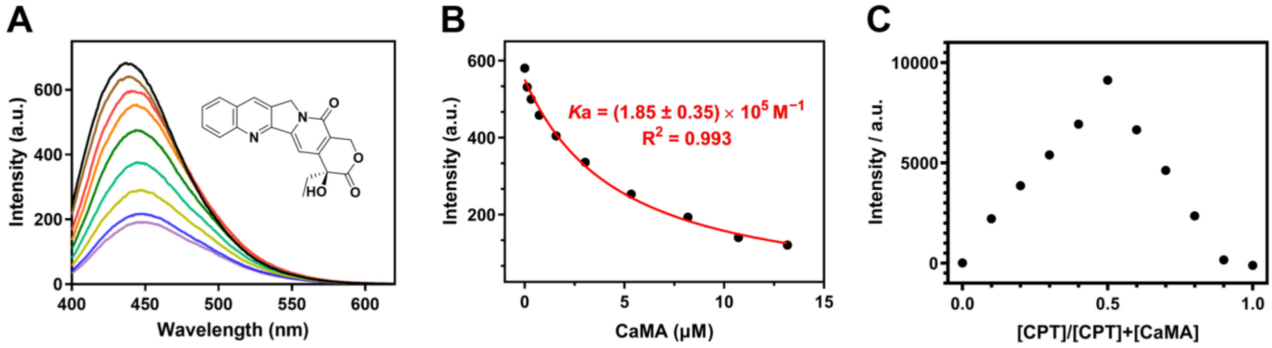

Figure S8. (A) Direct fluorescence titration of CPT (0.5 μM) with CaMA (up to 13.2 μM) in PBS buffer at 25 ˚C, λ_{ex}= 365 nm; (B) The associated titration curve at λ_{em} = 420 nm was fitted according to a 1:1 binding stoichiometry. (C) Job's plot for solutions of CPT and CaMA, [CPT] + [CaMA] = 2.0 μM.

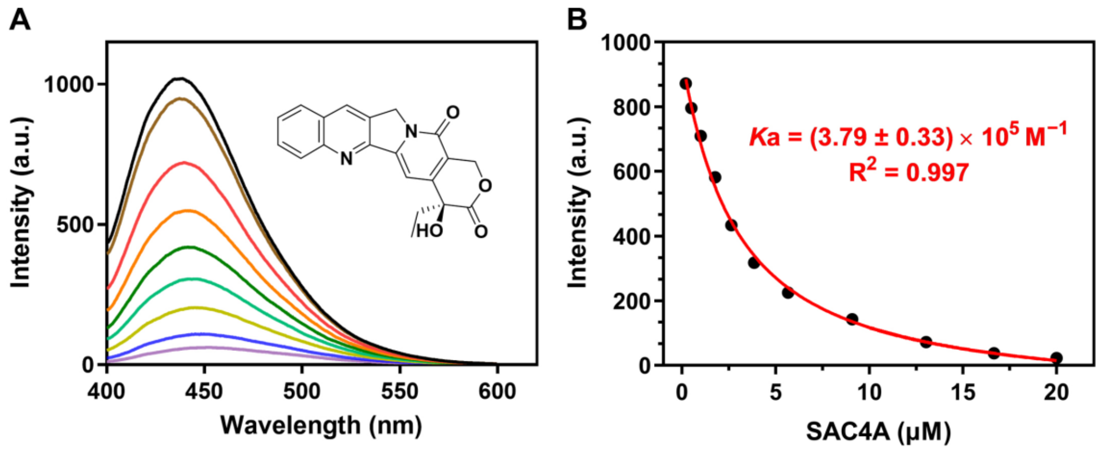

Figure S9. (A) Direct fluorescence titration of CPT (0.5 μM) with SAC4A (up to 20.0 μM) in PBS buffer at 25 ˚C, λ_{ex}= 365 nm; (B) The associated titration curve at λ_{em} = 420 nm was fitted according to a 1:1 binding stoichiometry.

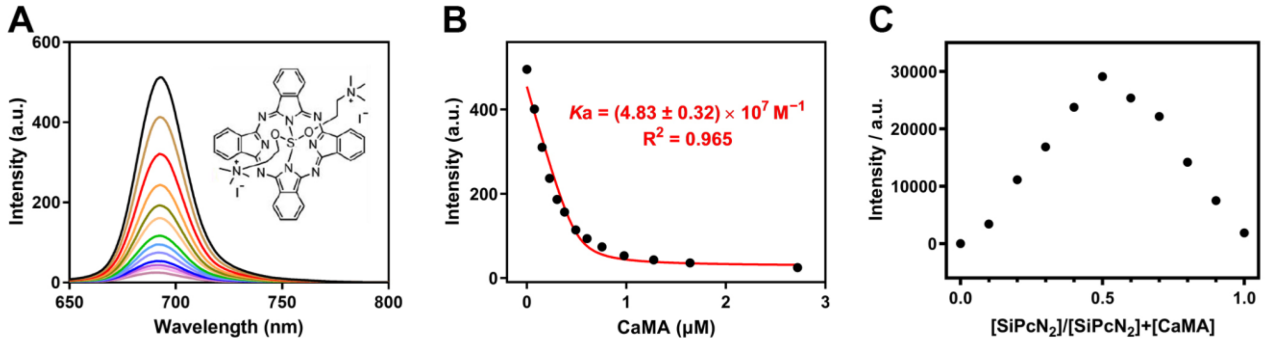

Figure S10. (A) Direct fluorescence titration of $SiPcN_2$ (0.5 μM) with CaMA (up to 2.72 μM) in PBS buffer at 25 ˚C, λ_{ex}= 610 nm; (B) The associated titration curve at λ_{em} = 690 nm was fitted according to a 1:1 binding stoichiometry. (C) Job's plot for solutions of $SiPcN_2$ and CaMA, [$SiPcN_2$] + [CaMA] = 2.0 μM.

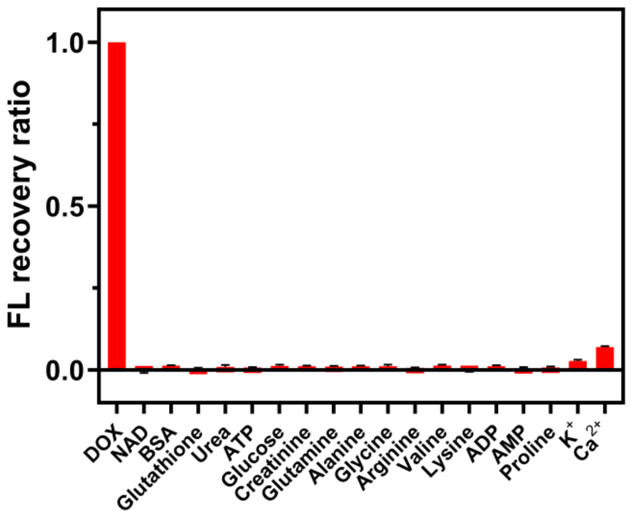

Figure S11. Fluorescence (FL) recovery ratio of CaMA-DOX in the present of various biologically species in the blood.

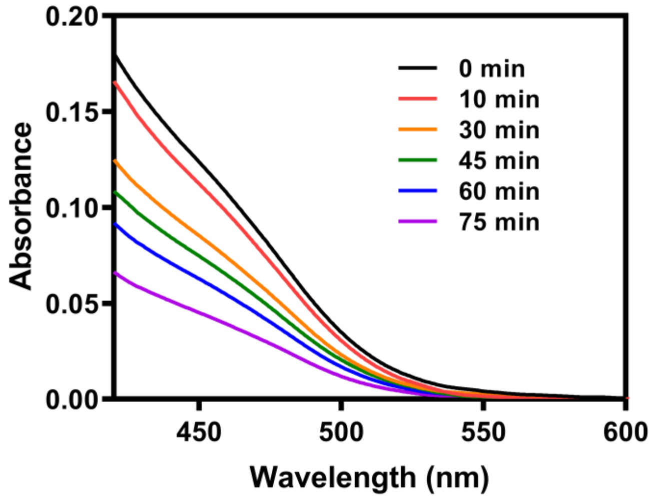

Figure S12. Absorbance spectra of CaMA (5.0 μM) as a function of time following addition of DT-diaphorase (1.0 μM) and NADPH (50 μM) in PBS (10 mM, pH 7.4) at 37 ˚C under hypoxic conditions.

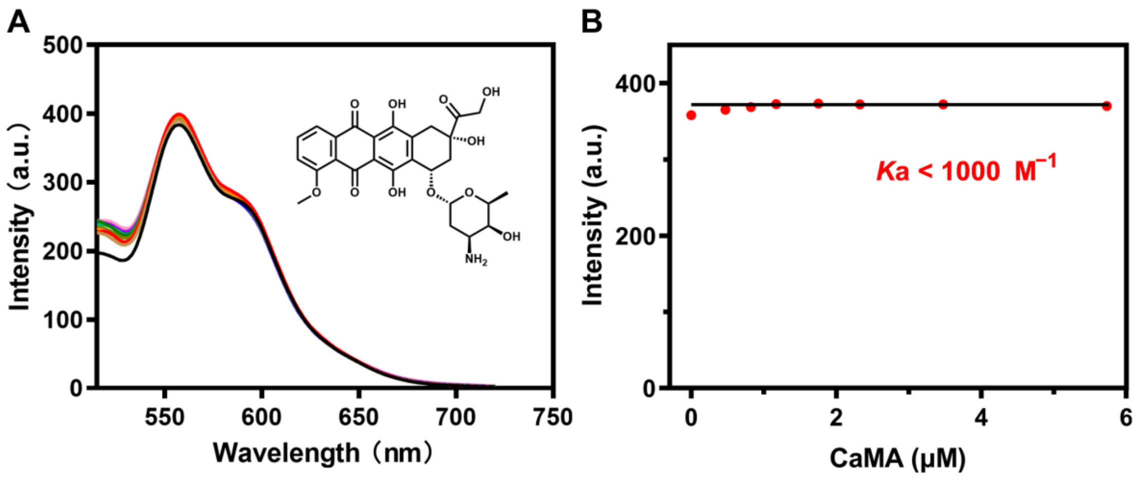

Figure S13. (A) Direct fluorescence titration of DOX (0.5 μM) with BSA-NH_2C4A (up to 5.73 μM) in PBS buffer at 25 ˚C, λ_{ex}= 497 nm; (B) The associated titration curve at λ_{em} = 555 nm was fitted according to a 1:1 binding stoichiometry.

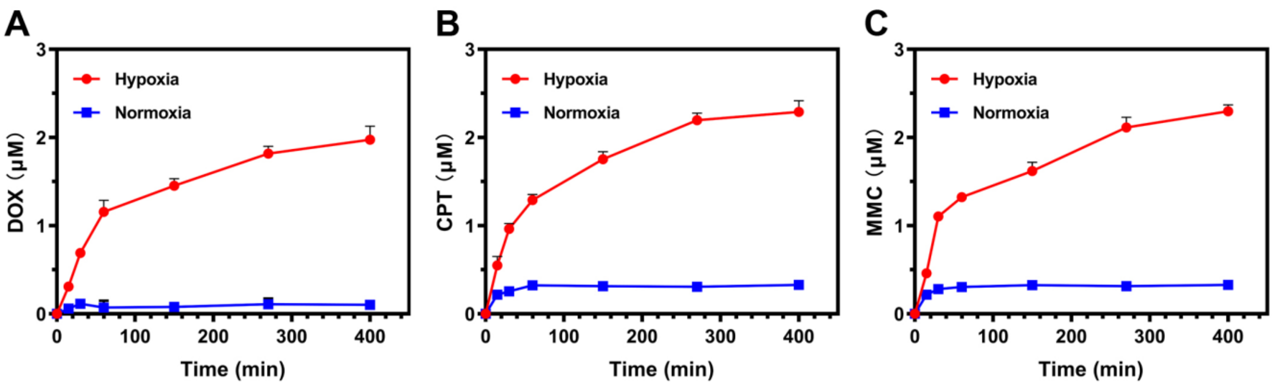

Figure S14. The release profiles of DOX (A), CPT (B) and MMC (C) from CaMA as the function of time following addition of DT-diaphorase and NADPH in PBS under different conditions.

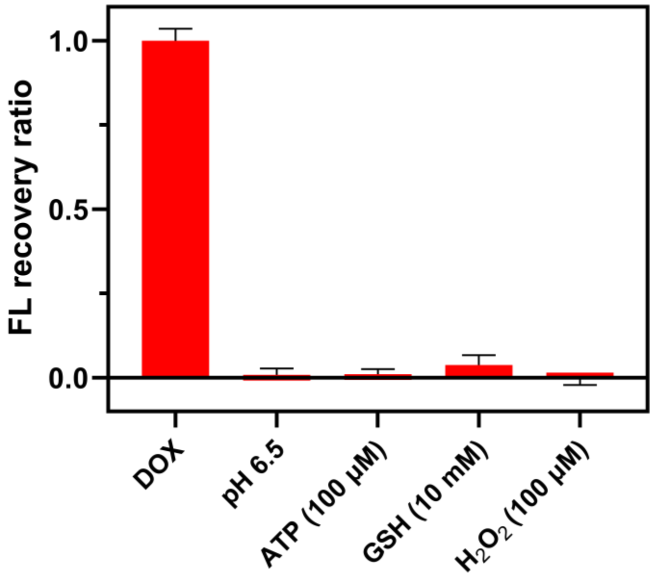

Figure S15. Fluorescence (FL) recovery ratio of CaMA-DOX in the present of various biologically species in the tumor.

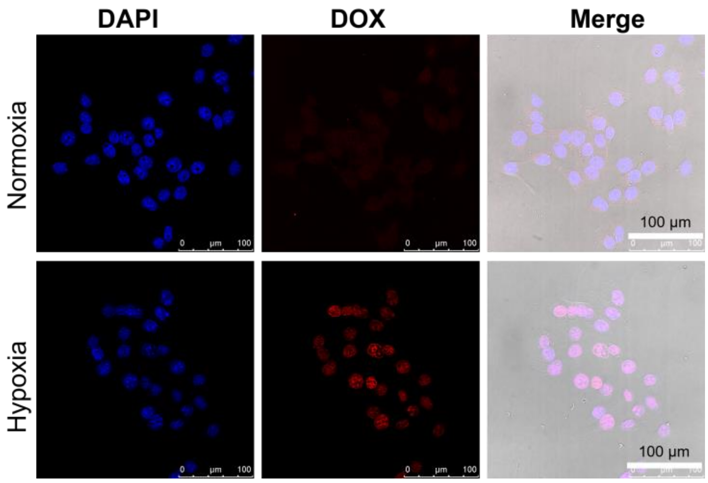

Figure S16. CLSM images of 4T1 cells after treatment with CaMA-DOX under normoxic or hypoxic conditions and stained by DAPI.

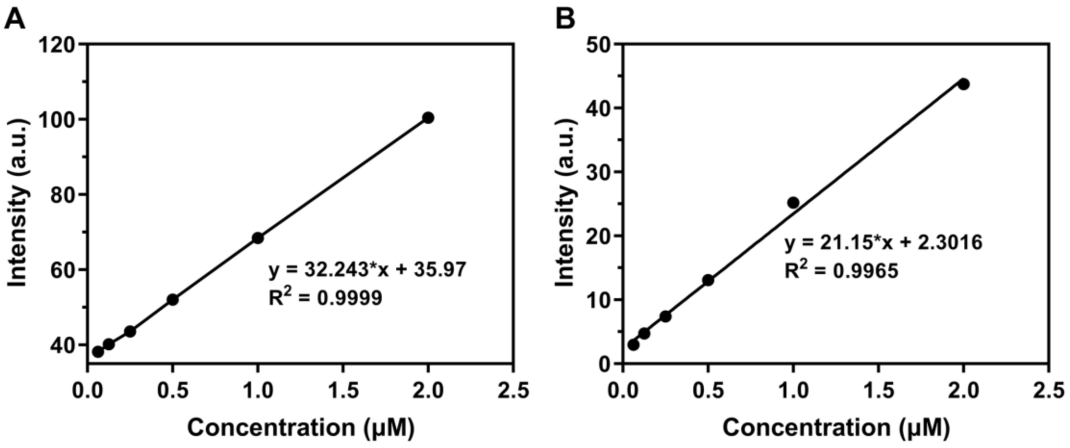

Figure S17. The standard curves of DOX (A) and CPT (B).

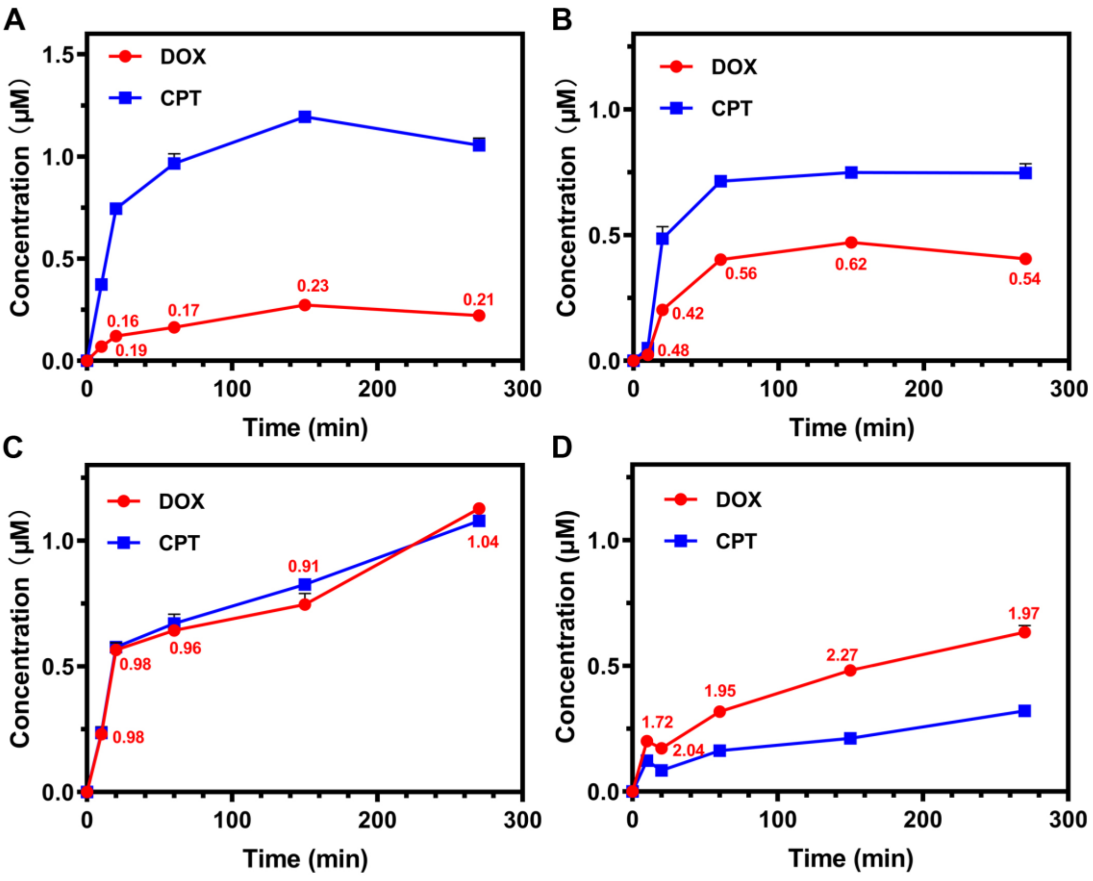

Figure S18. The release profiles of DOX and CPT from CaMA-DM with loading ratio between DOX and CPT at 0.2 : 1 (A), 0.5 : 1 (B), 1 : 1 (C) and 2 : 1 (D) as the function of time following addition of DT-diaphorase and NADPH in PBS under hypoxic condition.

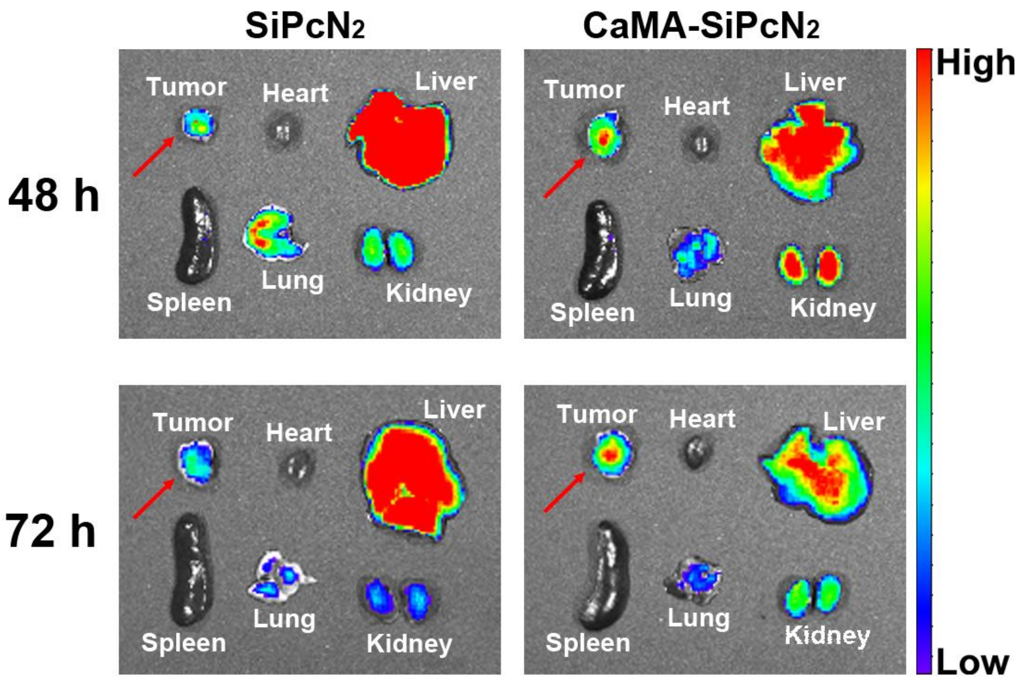

Figure S19. *Ex vivo* images of major organs from the mice treated with free $SiPcN_2$ and CaMA-$SiPcN_2$ at 48 and 72 h post-injective.

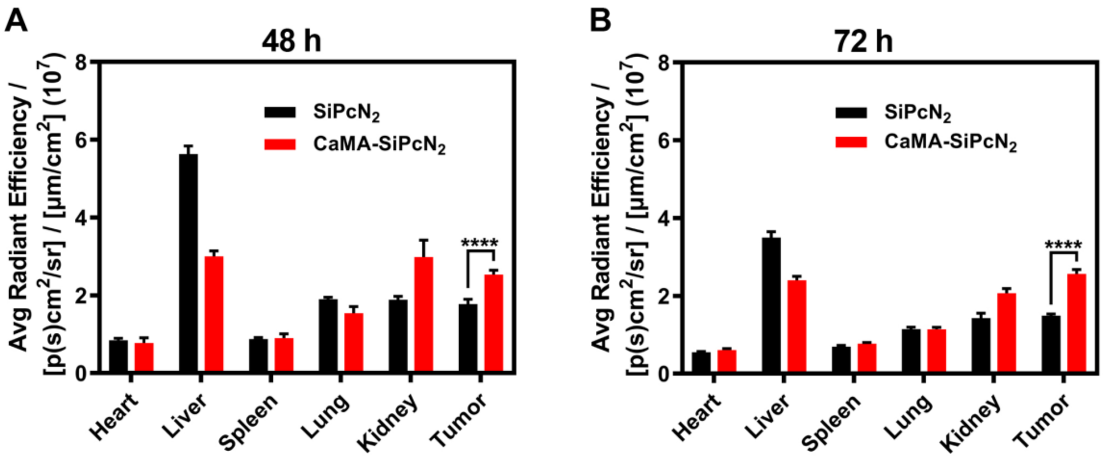

Figure S20. Quantitative analysis of accumulation in major organs of SiPcN2 and CaMA-SiPcN2 at 48 h (A) and 72 h (B), based on the fluorescence intensity from the *ex vivo* images.

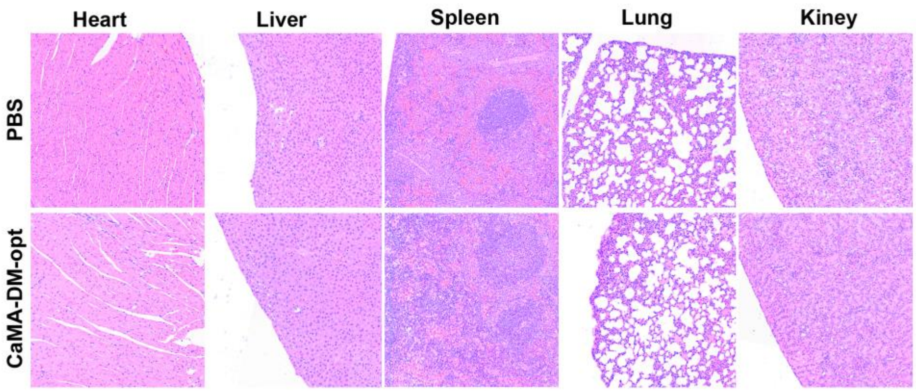

Figure S21. H&E stains of the major organs from mice treated with PBS and CaMA-DM-opt.

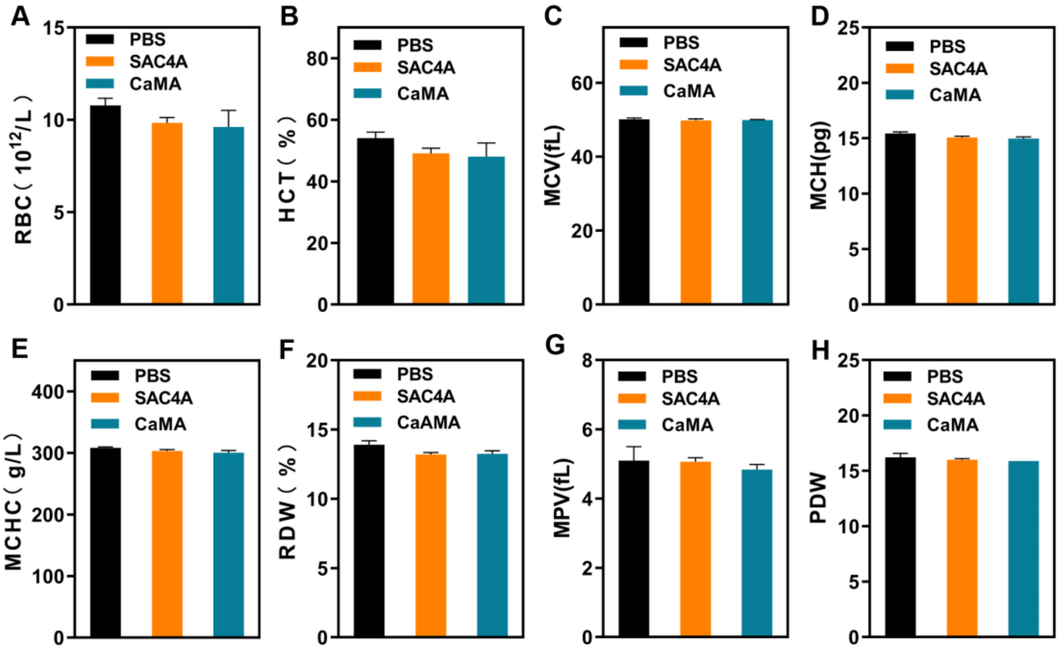

Figure S22. Safety evaluation of CaMA. A–H) red blood cell (RBC) (A), hematocrit (HCT) (B), mean corpuscular volume (MCV) (C), mean corpuscular hemoglobin (MCH) (D), mean corpuscular hemoglobin concentration (MCHC) (E), red blood cell volume distribution width (RDW) (F), mean platelet volume (MPV) (G) and platelet distribution width (PDW) (H) levels from mice treated with PBS, SAC4A, CaMA.

Reference

1. Zhang Z, Yue YX, Xu L, Wang Y, Geng WC, Li JJ, et al. Macrocyclic-Amphiphile-Based Self-Assembled Nanoparticles for Ratiometric Delivery of Therapeutic Combinations to Tumors. Adv Mater. 2021; 33: e2007719.
2. Guo D-S, Uzunova VD, Su X, Liu Y, Nau WM. Operational calixarene-based fluorescent sensing systems for choline and acetylcholine and their application to enzymatic reactions. Chem Sci. 2011; 2,1772.
